# Editorial: Phylogenomic discordance in plant systematics

**DOI:** 10.3389/fpls.2023.1308126

**Published:** 2023-10-27

**Authors:** Stefan Wanke, Susann Wicke

**Affiliations:** ^1^ Institute for Botany, Technische Universität Dresden, Dresden, Germany; ^2^ Departamento de Botánica, Instituto de Biología, Universidad Nacional Autónoma de México, Mexico City, Mexico; ^3^ Institute for Biology, Humboldt-Universität zu Berlin, Berlin, Germany

**Keywords:** gene tree, species tree, incomplete lineage sorting, plastid (chloroplast) DNA, phylogenetic discordance, phylogenomic analysis, DNA barcoding, reticulate evolution

In the *omics*-age of molecular systematics, entire genomes or genomic segments often contradict each other or the broader consensus of organismal relationships. Furthermore, this potentially conflicts with pre-*omics* phylogenetics, where true conflicts often remained undiscovered due to data limitations. Unlike earlier times when adding markers and taxa might have helped to address or even (superficially) resolve conflicts around phylogenetic hypotheses, incongruences arising from genome inferences remain challenging. Phylogenetic discordance can result from various evolutionary processes that lead to disparities between gene trees and species trees. This extends to genomic discordance, where organellar and nuclear genomes exhibit different coalescent paths or phenomena such as organelle capture. Advances in analytical methods enable the comparison of hundreds to thousands of loci across all plant genomes, offering a comprehensive view of phylogenomic complexity. This Research Topic explores high-throughput sequence-based phylogenomic studies that uncover discordant phylogenies. A total of 79 authors present a rich array of 14 original research articles focusing on phylogenomic studies based on high-throughput sequence data. These studies delve into the discordant phylogenies between, among, or within organellar genomes and the nuclear genome.

Through large-scale comparative analysis of over 3,600 plant plastomes, Yang et al. contribute to the growing body of studies that find potential issues with phylogenetic inference using plastid-only data, which suffer from saturation at third codon positions. Similarly, organellar phylogenomic analysis by Wu et al. of Poales, one of the largest monocot orders, attribute phylogenetic conflicts to potential ancient rapid radiation, advocating the integration of nuclear data to fully resolve relationships. In fact, a set of articles caution against overreliance on organellar genomes in resolving evolutionary relationships, due to their intrinsic limitations. Low mutation rates, extensive homoplasy, and lack of taxonomic coherence limit the utility of plastid genomes, for example in *Salix* spp. (Salicaceae), as Wagner et al. demonstrate through their analysis of shrub willow plastomes and comparing these to RAD sequencing-based data. They suggest nuclear data may better resolve biogeographical questions, as these reflect not just one coalescence line.

Nuclear genomes have their own challenges. Wu et al. found substantial phylogenetic conflicts within the plastid genomes of the Poales, as well as among the plastid, mitochondrial, and nuclear data, suggesting a complicated evolutionary history with rapid radiation and polyploidy, e.g. through hybridization. Such findings lend credence to calls by Jost et al. and Kandziora et al. for integrating evidence across genomic compartments. While organellar genomes have proven value in DNA-barcoding applications, resolving deep phylogenies may require more judicious data integration. Garrett et al. point out extensive gene tree conflicts in *Euphrasia* spp. (Orobanchaceae), limiting the utility of genome skimming for species identification. Such factors underscore the need for robust practices as phylogenomic datasets grow in scale. Complementing this topical complex, Hernández-Gutiérrez et al. advocate considering rate heterogeneity across loci and applying sorting approaches to mitigate its confounding effects. Thureborn et al. used the normalized quartet score (NQS) to assess gene tree discordance for the coffee family Rubiaceae, and Hodel et al. employed network analysis to examine phylogenetic discordance in their study of the apple tribe (Maleae, Rosaceae). The insights from these methodological approaches inform efforts to analyze phylogenomic datasets. Gene tree estimation and gene tree/species tree reconciliation practices also warrant scrutiny, with Kandziora et al. noting the potential of paralogy to mislead phylogenetic inference.

Resolving phylogenetic relationships within plant lineages where rapid diversification occurred millions of years ago can be particularly challenging for several reasons, incl. limited genetic variation, incomplete lineage sorting (ILS), hybridization, long branch attraction, limited fossil record, lack of informative characters, or complex evolutionary processes such as adaptive radiation, where species rapidly adapt to exploit different ecological niches. These processes can result in intricate patterns of diversification that are challenging to unravel. Among such phylogenetically challenging groups of plants are sages (*Salvia* spp., Lamiaceae), comprising approximately a thousand species. Rose et al. and Lara-Cabrera et al. both used a combination of nuclear and plastid data obtained from hybrid enrichment and off-target plastome sequences to infer gene and species phylogenies by Bayesian and Maximum Likelihood (ML) multispecies coalescent-based approaches. To examine the concordance and discordance among nuclear loci and between the nuclear and plastid genomes in detail, simulations were run to test whether ILS underlies the phylogenetic discordance (Rose et al.) and to infer the robustness of inferences in light of varying extents of missing data (Lara-Cabrera et al.). Together, these studies provide a well-supported backbone species tree of *Salvia* spp. across phylogenetic scales and genomes, suggesting that past difficulties in inferring relationships may have been caused by a combination of uninformative markers, ILS, and horizontal gene flow.

ILS arising from rapid radiations is also identified as major potential driver of phylogenomic discordance by Zheng et al. in their work on the *Quercus franchetii* complex (Fagaceae) spanning the Himalaya region since the Oligocene. They suggest that tectonic shifts and environmental heterogeneity have promoted allopatric speciation, restricting gene flow. This could have increased the chance of ILS, although the hypothesis of an ancient rapid diversification in the group remains to be tested. Likewise, Hernández-Gutiérrez et al. find short branches and incongruent relationships between Malvaceae lineages, indicating potential ILS during diversification. Using triplet analysis the study found that the signal of ILS can be obscured by even low levels of introgression. This underscores the need for robust methods like gene tree sorting and topology weighting, applied by Jost et al. and Kandziora et al. in Piperales and *Loricaria* (Asteraceae), respectively. Such approaches can provide greater confidence in elucidating whether ILS alone or complex factors underlie phylogenetic discordance.

A predominant theme emerging is the potential role of reticulate evolutionary processes like hybridization and introgression as contributors to phylogenomic discordance. Hernández-Gutiérrez et al. present evidence of introgression contributing to discordance on top of ILS-related conflicts between subfamilies in Malvaceae. In contrast, Liu et al. in their study on the *Pedicularis siphonantha* complex (Orobanchaceae), endemic to Southwest China, implicate ancient hybridization events in shaping the topological conflicts observed between nuclear and plastid phylogenies. Similarly, Hsieh et al., through their analysis of 93 plastid genomes representing all genera of Berberidaceae, suggest that ancient hybridization between diverging lineages gave rise to intermediate genera like *Alloberberis*. They note substantial sequence variation in plastid markers among species, thereby highlighting plastomic fluidity. While these specific cases lend evidence for hybridization’s influence, its pervasiveness and evolutionary importance across diverse plant families require further investigation through rigorous assessments to avoid overstating its role.

In terms of implications, Hsieh et al., Rose et al., and Garrett et al. note that extensive phylogenetic discordance poses challenges for taxonomy, species delimitation, and DNA barcoding efforts in diverse plant groups. Extended barcodes may have limited utility in taxa exhibiting high gene tree conflicts. Hernández-Gutiérrez et al. posit that resolving deep phylogenetic uncertainties may require moving beyond just amassing larger genomic datasets, to focusing on data quality and model adequacy. Meanwhile, Yang et al. suggest dense taxon sampling may not always improve phylogenetic accuracy in the face of pervasive ILS. Such perspectives serve as important reminders that more data does not automatically equate to simpler evolutionary interpretations. It also (re)opens an exciting debate on how plant classification can develop under coexisting phylogenetic hypotheses that potentially arise from (currently) unresolvable topological conflicts among large sets of gene trees.

In synthesizing these findings, a central theme emerges: the widespread occurrence of phylogenetic discordance, arising from a complex interplay of biological and methodological factors. Factors such as reticulate evolution, incomplete lineage sorting, and rapid radiations all contribute to the intricate tapestry of evolutionary histories. To move forward, we must prioritize the development of robust comparative methods and study designs that harness the power of genomic data. It is crucial to approach phylogenetic conflicts with care, integrating evidence from various data types while acknowledging the heterogeneity among (sub-)genomic regions. Embracing the intricacies unveiled through phylogenomics grants us deeper insights into the mechanisms driving plant diversity. However, this expanding body of knowledge should also foster humility as we increasingly appreciate the multifaceted nature of evolutionary narratives.

## Author contributions

SWa: Conceptualization, Writing – original draft. SWi: Conceptualization, Writing – original draft.

